# Vitality tracking in Kenyan adults using wearable devices and ecological momentary assessments

**DOI:** 10.1002/alz70856_103471

**Published:** 2025-12-24

**Authors:** Karen Blackmon, Benard Alaka, Catherine Bikeri Onyancha, Litha Musili, Raechel Kamau, Cyprian M Mostert, Mansoor Saleh, Zul Merali, Chinedu T Udeh‐Momoh, Thomas Thesen

**Affiliations:** ^1^ Geisel School of Medicine at Dartmouth, Hanover, NH, USA; ^2^ Dartmouth Hitchcock Medical Center, Lebanon, NH, USA; ^3^ Brain and Mind Institute, Aga Khan University, Nairobi, Kenya; ^4^ Aga Khan University, The Brain and Mind Institute, Nairobi, Kenya; ^5^ Global Brain Health Institute, Trinity College Dublin, University of Dublin, Ireland; ^6^ Aga Khan University, The Brain and Mind Institute, Nairobi, Nairobi, Kenya; ^7^ FINGERS Brain Health Institute, Solna, Stockholm, Sweden; ^8^ Global Brain Health Institute, University of California, San Francisco, NC, USA; ^9^ Division of Public Health Sciences, Wake Forest University, School of Medicine, Winston‐Salem, NC, USA; ^10^ The Geisel School of Medicine at Dartmouth, Hanover, NH, USA

## Abstract

**Background:**

Vitality is essential to healthy aging but is elusive to define and measure. Dynamic capture of fluctuations in vitality can uncover abnormal trajectories that could be a harbinger of frailty in older adults. Yet, these fluctuations are missed by static and/or sparse sampling strategies. To address the need for dynamic and high‐density capture of vitality in older adults, we developed a passive sensing and active probing digital platform using wearable devices and smartphones. We deployed this paradigm in older Kenyan adults and evaluated its potential for use in research on the dynamics of accelerated aging.

**Method:**

Cognitively unimpaired (CU) Kenyan adults ≥ 35 years of age (*N* = 79) were provided with the Fitbit Inspire 3 device to sense heart rate, sleep, and physical activity over the course of 12 months. At baseline, they completed neurocognitive screening to confirm CU status. A novel 10‐item Vitality Index (VI) was developed and delivered weekly through a mobile phone app to assess fluctuations in health, strength, energy, pain, sleep, mood, and focus, alongside a brief spatial working memory task. Here, we evaluate adherence, internal consistency, test‐retest reliability, and convergent validity of the VI against gold standard measures of depression (PHQ‐9), anxiety (GAD‐7), and flourishing.

**Results:**

Data from 79 participants [53 m / 25 f /1 intersex; median education: 12 y; mean age (sd)=49 (9.7) y; age range: 35‐74 y] show that 97% of participants wear their device at least 95% of the time. Weekly VI completion averages 85% and weekly spatial memory task completion averages 96%. The VI shows good internal consistency (week 1 α = 0.75) and test‐retest reliability (r=0.73; *p* <0.001). VI is positively negatively correlated with depression (r=‐0.34; *p* <0.01) and anxiety (r=‐0.37; *p* <0.01) but positively correlated with flourishing (r=0.27; *p* <0.01).

**Conclusion:**

A high degree of compliance with wearable sensors and weekly assessments, and sound psychometrics, demonstrates the feasibility of our vitality tracking paradigm in older Kenyan adults. Future analyses will uncover biopsychosocial risk factors that contribute to irreversible loss of vitality. Findings will provide essential information for future interventional studies that aim to trial behavioral and pharmaceutical approaches to rescuing vitality in at‐risk adults.

